# Systemic immune inflammatory index is an independent predictor for the requirement of decompressive craniectomy in large artery occlusion acute ischemic stroke patients after mechanical thrombectomy

**DOI:** 10.3389/fneur.2022.945437

**Published:** 2022-09-27

**Authors:** Wen-Cai Li, Yun-Xiang Zhou, Gang Zhu, Kai-Liang Zeng, Hai-Yong Zeng, Jian-Sheng Chen, Yi-Fan Deng, Zhong-Zong Qin, Hong-Hai Luo

**Affiliations:** ^1^Department of Neurosurgery, Huizhou Central People's Hospital, Huizhou, China; ^2^Department of Neurosurgery, Affiliated Hospital of Guilin Medical University, Guilin, China

**Keywords:** systemic immune-inflammation index, large artery occlusive acute ischemic stroke, mechanical thrombectomy, decompressive craniectomy, nomogram

## Abstract

**Background and purpose:**

Following mechanical thrombectomy (MT), patients with large artery occlusive acute ischemic stroke (LAO-AIS) often have cerebral herniation due to its complications, resulting in poor prognosis. Decompressive craniectomy (DC) can markedly improve patient prognosis. This study aimed to verify the predictive value of clinical parameters such as the systemic immune-inflammatory index (SII) for DC in patients with LAO-AIS after MT.

**Methods:**

Clinical data of a total of 173 patients with LAO-AIS treated with MT between January 2020 and January 2022 were retrospectively analyzed. Patients receiving DC were grouped into an experimental group or a control group (no DC). The patients were randomly divided into the training set (*n* = 126, 75%) and validation set (*n* = 43, 25%). Multivariate logistic regression was used to construct a nomogram predictive model.

**Results:**

The SII value in the experimental group (median: 2851.1×10^9^/L) was significantly higher than that in the control group (median: 1898.6 × 10^9^/L) (*P* = 0.019). Receiver operating characteristic (ROC) analyses showed that the best cutoff value of the SII was 2505.7 × 10^9^/L with a sensitivity of 55%, a specificity of 75.8%, and an area under the curve (AUC) of 0.649. Multivariate logistic regression indicated that the SII was an independent predictor for performing DC in patients with LAO-AIS after MT (OR = 3.579, 95% CI = 1.360–9.422, *P* = 0.01). The AUC was 0.728 in the training set and 0.583 in the validation set. The average error of the calibration curve was 0.032 in the training set and 0.023 in the validation set. The average error was relatively small and consistent in the training set and validation set. The C-index of the nomogram was 0.804 suggesting good accuracy.

**Conclusions:**

The SII at admission is an independent predictor for the requirement of DC in patients with LAO-AIS after MT. The SII-based nomogram helps doctors make decisions on whether DC is needed timely and rationally, and thereby may improve the prognosis of these patients.

## Introduction

Globally, and especially in low- and middle-income countries, stroke is the leading cause of death and disability, and most patients who survive the acute phase of stroke show varying degrees of neurological deficits (1, 2). Large arterial occlusion (LAO) has become one of the most important causes of acute ischemic stroke (AIS) worldwide and is closely related to the poor prognosis of AIS patients (3). In the past 10 years, endovascular treatment of large-arterial occlusion acute ischemic stroke (LAO-AIS) has achieved good progress (4). For such patients, mechanical thrombectomy (MT) is one of the main treatment approaches. MT shows clear efficacy in recanalization if used within 6 h after the onset of LAO-AIS, and its complete recanalization rate is ~50% (5–7). Brain reperfusion injury is the most serious complication after MT and mainly manifests as contrast extravasation (CE) and hemorrhagic transformation (HT) (8). CE and HT are closely associated with poor prognosis, with a mortality rate close to 50%. The increase and imbalance of intracranial pressure caused by CE or HT can induce brain herniation, which leads to severe neurological deterioration and even death (9).

Decompressive craniectomy (DC), as a salvage operation, can treat patients with refractory intracranial hypertension and early brain herniation by relieving intracranial pressure (10). The current opinion is that once brain herniation is detected by imaging, DC can be performed as soon as possible without waiting for neurological deterioration (11). A Brazilian study showed that early DC could significantly improve the prognosis of AIS patients (12). Bruno Askiel et al. (13) proposed that patient age and midline shift after DC could predict neurological outcomes in post-DC patients. Oliveira BDD et al. (14) stated that ultrasound monitoring of the optic nerve sheath diameter can identify severe intracranial hypertension in patients with large vessel occlusion, and this parameter can be used as a predictive factor for DC. However, it is still necessary to find more convenient and practical predictors for the requirement of DC to allow doctors to make decisions more quickly and improve patient outcomes.

In recent years, an increasing number of studies have focused on the inflammatory response after stroke. Inflammation is thought to be involved in recovery initiation and repair processes after stroke, yet some aspects of the inflammatory response may instead have detrimental effects in stroke patients (15). The systemic immune-inflammation index (SII), a new type of inflammatory index, is calculated by combining three immune inflammatory cells—lymphocytes, neutrophils, and platelets—and fully reflects the inflammation status (16). A high SII level increases the risk of stroke in patients with hypertension and affects patients' prognosis (17). Therefore, the purpose of our study was to calculate the SII according to the first routine blood test at admission, construct a model for the prediction of DC requirements, and verify the predictive value of the SII to provide a tool for the selection of DC in patients with LAO-AIS after MT.

## Methods

### Patient selection

We retrospectively analyzed the data of patients who were diagnosed with LAO-AIS and underwent MT in Huizhou Central People's Hospital from January 2020 to January 2022. We included patients who met the following criteria: (1) those who were admitted within 24 h after disease onset, with symptoms and signs of neurological dysfunction and confirmed with LAO-AIS by cranial CT angiography (CTA) or whole cerebral angiography; (2) those that met the MT criteria and received MT in our hospital. The indications of mechanical thrombectomy are as follows: AIS with National Institutes of Health Stroke Scale (NIHSS) ≥ 6; Cerebral infarction caused by large artery occlusion of anterior circulation; Alberta Stroke Program Early CT Score (ASPECTS) ≥ 6; CT or MRI ruling out ICH; and groin puncture for MT within 6 h of symptom onset (18). It is worth mentioning that the surgical indications for mechanical thrombectomy in wake-up stroke patients are defined as follows:(1) Patients with anterior circulation large vessel occlusion within 6–16 h from the last normal state of the patient and meeting the enrollment criteria of DAWN or DEFUSE-3 study. (2) Patients with anterior circulation large vessel occlusion within 6–24 h from the last normal state of the patients and meeting the enrollment criteria of the DAWN study (19); and those that were (3) >18 years of age. The following patients were excluded: (1) those that did not complete routine blood tests at admission and lacked required laboratory data; (2) those with a history of cerebral infarction, and MRS ≥2 points; (3) those with a history of infection within 2 weeks; (4) those complicated by hematological diseases; (5) those undergoing administration of immunosuppressive drugs; (6) those with a history of malignant tumor or autoimmune disease; or (7) patients with posterior circulation occlusion.

This study was approved by the Ethics Committee of Huizhou Central People's Hospital (kyll2022029) and was conducted in accordance with the Declaration of Helsinki. As this was a retrospective study, the Ethics Committee approved the application for a waiver of signed informed consent. To protect patient privacy, this study anonymized the identifiable information of the enrolled patients.

### Data collection

We collected baseline clinical data of all patients, including age, gender, and medical history of coronary heart disease, hypertension, diabetes, smoking and drinking, atrial fibrillation, and hyperlipidemia. Hypertension was defined as a prior history of hypertension with systolic/diastolic blood pressure ≥140/90 mmHg at admission. Diabetes was defined as a history of diabetes, fasting blood glucose (FBG) ≥7 mmol/L, and random blood glucose ≥11.1 mmol/L at admission. Coronary heart disease was defined as a history of coronary heart disease. Atrial fibrillation was defined as a history of atrial fibrillation or an ECG diagnosis at admission.

We collected the routine blood, blood biochemistry, hemostatic function, and other serological test results of the patients obtained in the emergency department or within 1 day of admission. White blood cell count in the range of (4–10) × 10^9^/L was defined as normal, otherwise abnormal. Neutrophil count in the range of 2–7 × 10^9^/L was defined as normal, otherwise abnormal. The absolute count of lymphocytes in the range of 0.8–3.5 × 10^9^/L was defined as normal, otherwise abnormal. The normal range of platelets was (100–300) × 10^9^/L. Abnormal test results also included: PT > 14 s, APTT > 37s, D-D dimer > 500 ng/ml, FIB > 4 g/L, LDL > 3.1 mmol/L, TG > 1.7 mmol/L, serum potassium < 3.5 mmol/L, serum calcium < 2.11 mmol/L and albumin < 40 g/L. The patients' inflammation was evaluated by collecting and calculating the platelets, neutrophils, and lymphocytes in the blood during routine examination of the patients when they were in the emergency department or just admitted to the hospital. The calculation formula is SII = platelets × neutrophils/lymphocytes.

### Outcome event

In this study, whether patients underwent DC was regarded as the outcome event. DC was defined as a positive outcome, and no DC was defined as a negative outcome. Indications for DC included: (1) Patients with massive cerebral infarction had early symptoms of a cerebral hernia within 48 h of onset (Clinical manifestations such as disturbance of consciousness, Cushing reaction, unilateral mydriasis, Centerline offset ≥5 mm, Ipsilateral lateral ventricle compression, Sulci cistern compression); (2) After active medical treatment, there are still obvious manifestations of intracranial hypertension or progressive deterioration of neurological function (GCS score decreased ≥1 or new mydriasis or light reflex changes or new focal motor function defects); (3) For patients with large-area cerebral infarction in the middle cerebral artery region, EDEMA score ≥3 points or modified EDEMA score ≥6 points (19, 20).

### Statistical analysis

Patients undergoing DC were classified as the experimental group, otherwise, the control group. The data were tested for normality using SPSS 23 (SPSS Inc., Chicago, IL, USA). The continuous data which conformed to the normal distribution were expressed as mean ± standard deviation, and the *t*-test was used for inter-group comparisons; otherwise, the continuous data were expressed as the median and quartile, and the rank sum test was used for inter-group comparisons. The count data were expressed as percentage *n* (%), and the chi-square test was used for inter-group comparison. If the expected frequency of more than 20% of cells in the contingency table was <5, Fisher's test was used. Baseline data of the two groups were compared to assess their comparability.

The *t*-test or rank-sum test was used to compare the difference in SII between the experimental and control group. GraphPad Prism 8.3 was used to draw a bar plot of the SII values of the two groups and was also used to plot the receiver operating characteristic (ROC) curves to evaluate the predictive value of the SII for DC. The cutoff value of SII was determined according to the ROC curve, and the cutoff value was used to divide SII into the high and low groups. Univariate logistic regression was first conducted to identify factors associated with adverse outcomes, and odds ratios (ORs) and 95% CIs were calculated. Significant factors in the univariate analysis were included in the multivariate logistic stepwise regression analysis to identify independent predictors. All tests were two sided and a *P* < 0.05 was considered statistically significant. Significant factors in the multivariate regression and common clinical indices were used to construct a nomogram prediction model and plotted by R studio (version 4.1.0). The ROC curve and area under the curve (AUC) were used to assess the predictive performance of the nomogram model. The goodness of fit of the nomogram was assessed by the calibration curve and average error. The C-index was calculated to evaluate the accuracy of the nomogram.

## Results

### Baseline characteristics

**Figure 1:**
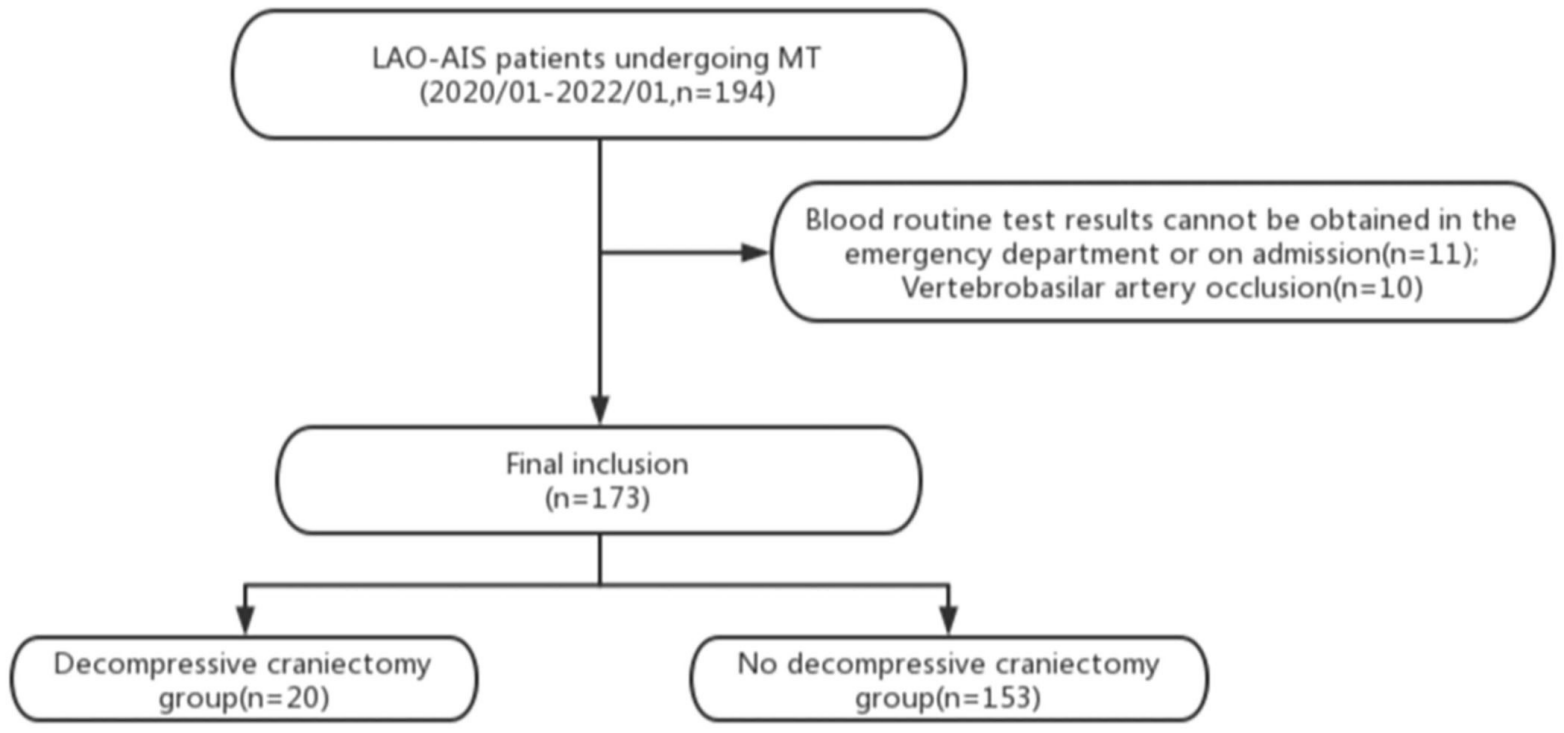
Flow chart of this study. LAO-AIS, large-arterial occlusion acute ischemic stroke; MT, mechanical thrombectomy.

We collected the clinical data of a total of 194 patients with LAO-AIS undergoing MT. Eleven patients did not undergo routine blood tests at admission and lacked the required laboratory data, 10 patients were excluded due to vertebrobasilar artery occlusion. These patients were excluded, and the remaining 173 patients were included in the analysis. Twenty and 153 patients were included in the experimental group and control group, respectively (Figure 1). The experimental group consisted of 13 men and 7 women with an average age of 56.1 ± 5.6 years. In the experimental group, the left side was involved in 11 patients and the middle cerebral artery was the responsible artery in 11 patients. The control group consisted of 105 males and 48 females with an average age of 56.6 ± 9.2 years. In the control group, 76 patients had lesions on the left side and the middle cerebral artery was the responsible artery in 99 patients. The average systolic blood pressure and diastolic blood pressure in the 173 patients were 143.8 ± 26.3 mmHg and 85.9 ± 16.3 mmHg at admission, respectively, and 52% of patients had a history of hypertension. In addition to hypertension, patients who had a history of atrial fibrillation, diabetes, hyperlipidemia, smoking, and alcoholism accounted for 22, 19.1, 26.2, 39.3, and 14.5%, respectively. There were no significant differences in the baseline characteristics between the two groups (Table 1), indicating comparability between these two groups.

**Table 1:** Baseline table.

		**Total**	**Experience group**	**Control group**	***P*-value**
			**(*n* = 20)**	**(*n* = 153)**	
Age (years)		56.9 ± 8.9	56.1 ± 5.6	56.6 ± 9.2	0.799
Gender (*n*)	Male	118 (68.2%)	13 (7.5%)	105 (60.7%)	0.743
	Female	55 (31.8%)	7 (4.0%)	48 (27.7%)	
Systolic BP (mmHg)		143.8 ± 26.3	137.6 ± 35.5	144.6 ± 24.9	0.268
Diastolic BP (mmHg)		85.9 ± 16.3	85.7 ± 26.6	85.9 ± 14.5	0.976
Hypertension (*n*)	Yes	90 (52.0%)	8 (4.6%)	82 (47.4%)	0.252
	No	83 (48.0%)	12 (6.9%)	71 (41.0%)	
Atrial fibrillation (n)	Yes	38 (22.0%)	3 (1.7%)	35 (20.2%)	0.424
	No	135 (78.0%)	17 (9.8%)	118 (68.3%)	
Diabetes (*n*)	Yes	33 (19.1%)	4 (2.3%)	29 (16.8%)	0.911
	No	140 (80.9%)	16 (9.2%)	124 (71.7%)	
Hyperlipidemia (*n*)	Yes	48 (26.2%)	7 (4.0%)	37 (21.4%)	0.296
	No	135 (73.8%)	13 (7.5%)	116 (67.1%)	
Smoke (*n*)	Yes	68 (39.3%)	7 (4.0%)	61 (35.3%)	0.678
	No	105 (60.7%)	13 (7.5%)	92 (53.2%)	
Alcoholism (*n*)	Yes	25 (14.5%)	2 (1.2%)	23 (13.3%)	0.547
	No	148 (85.5%)	18 (10.4%)	130 (75.1%)	
Lesion Site (*n*)	Left	86 (47.0%)	11 (6.4%)	76 (43.9%)	0.654
	Right	97 (53.0%)	9 (5.2%)	77 (44.5%)	
Responsible Artery (*n*)	Middle Cerebral Artery	110 (63.6%)	11 (6.4%)	99 (57.2%)	0.396
	Internal Carotid Artery	63 (36.4%)	9 (5.2%)	54 (31.2%)	

### Factors associated with DC in patients with LAO-AIS after MT

**Figure 2:**
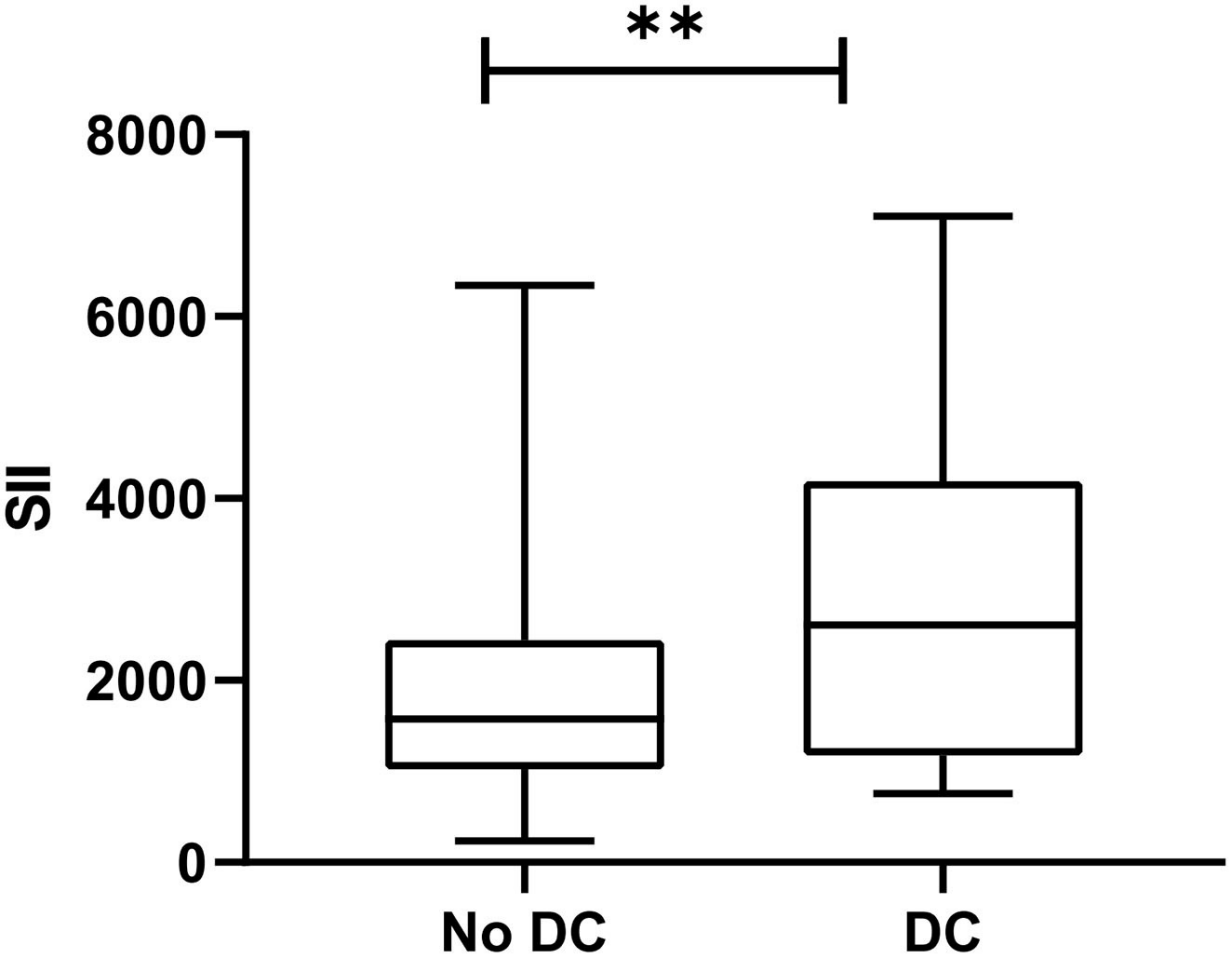
Comparison of SII between experimental group and control group. *p < 0.05.

**Figure 3:**
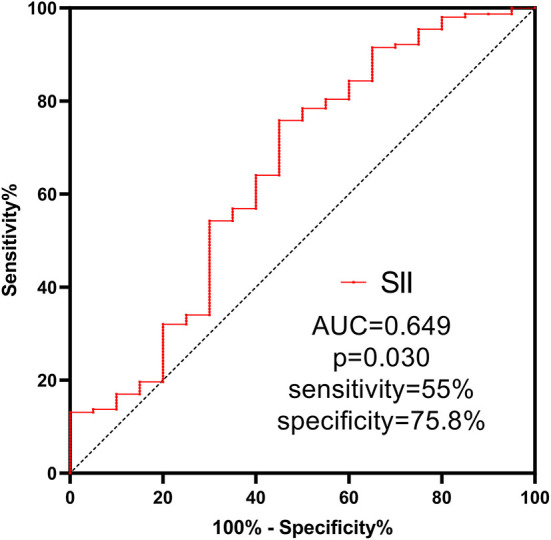
ROC curve of SII.

The SII value in the experimental group (median: 2851.1 × 10^9^/L) was significantly higher than that in the control group (median: 1898.6 × 10^9^/L) (*P* = 0.019, Figure 2). ROC analysis indicated that the best cutoff value of SII was 2505.7 × 10^9^/L, with a sensitivity of 55% and specificity of 75.8%. The AUC was.649 suggesting that the SII had a certain value in predicting DC (Figure 3). In order to further understand the relationship between the SII and DC, we divided the 173 patients into the high SII group and low SII group according to the ROC cutoff value (SII > 2505.7 × 10^9^/L, *n* = 125; SII ≤ 2505.7 × 10^9^/L, *n* = 48). Univariate analysis showed that hypokalemia and high SII were associated with DC. Multivariate analysis demonstrated that high SII was an independent risk factor for DC in patients with LAO-AIS after MT (OR = 3.579, 95% CI = 1.36–9.422, *P* = 0.01) (Table 2).

**Table 2:** Binary logistic regression analysis predicting outcome.

**Variable**		**Univariate analysis**			**Multivariate analysis**	
		**Patients**	**OR (95% CI)**	**Log-rank P**	**OR (95% CI)**	**Log-rank P**
Gender (*n*)				0.743		
	Male	118	1			
	Female	55	1.178 (0.442–3.139)			
Age (years)				0.847		
	>55	83	1			
	≤55	90	0.913 (0.359–2.318)			
Hypertension (*n*)				0.257		
	Yes	90	1			
	No	83	0.577 (0.223–1.492)			
Atrial fibrillation (*n*)				0.428		
	Yes	38	1			
	No	135	0.595 (0.165–2.149)			
Hyperlipidemia (*n*)				0.300		
	Yes	44	1			
	No	129	1.688 (0.627–4.546)			
Diabetes (*n*)				0.911		
	Yes	33	1			
	No	140	1.069 (0.332–3.437)			
Smoke (*n*)				0.675		
	Yes	68	1			
	No	105	0.812 (0.307–2.151)			
Alcoholism (*n*)				0.550		
	Yes	25	1			
	No	148	0.628 (0.136–2.891)			
PT (s)				0.849		
	≤14	98	1			
	>14	72	0.909 (0.340–2.432)			
	Missing	3				
APTT (s)				0.102		
	≤37	111	1			
	>37	59	0.343 (0.095–1.236)			
	Missing	3				
D-D (ng/ml)				0.209		
	≤500	40	1			
	>500	131	2.643 (0.581–12.027)			
	Missing	2				
FIB (g/L)				0.399		
	≤4	134	1			
	>4	39	1.558 (0.556–4.370)			
LDL (mmol/L)				0.917		
	≤3.1	106	1			
	>3.1	55	1.058 (0.369–3.030)			
	Missing	12				
K (mmol/L)				0.044		0.078
	≥3.5	121	1		1	
	<3.5	52	2.643 (1.026–6.804)		2.395 (0.907–6.324)	
Ca (mmol/L)				0.155		
	≥2.11	127	1			
	<2.11	46	2.018 (0.767–5.305)			
Triglyceride (mmol/L)				0.055		
	>1.7	22	1			
	≤1.7	131	2.018 (0.767–5.305)			
	Missing	20				
Albumin (g/L)				0.460		
	<40	96	1			
	≥40	68	1.476 (0.525–4.149)			
	Missing	9				
SII (× 109/L)				0.006		0.010
	>2505.7	125	1		1	
	≤2507.7	48	3.832 (1.474–9.963)		3.579 (1.360–9.422)	

### Predictive nomogram development

**Figure 4:**
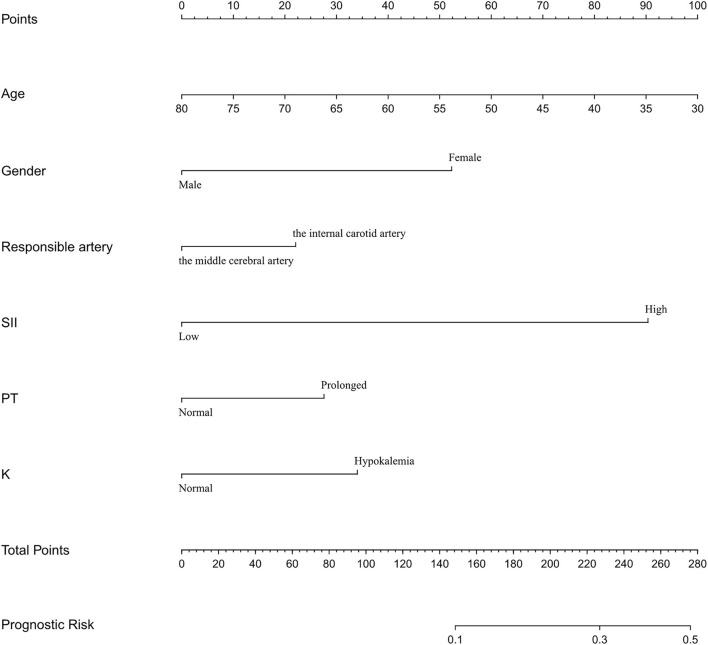
Nomogram of clinical data.

We then constructed a nomogram model for the prediction of DC. We incorporated the SII and common clinical indices into the nomogram. The patients were randomly divided into the training set (*n* = 126) and validation set (*n* = 43). Age, sex, SII, serum potassium, responsible artery, and PT were included in the binary logistic regression and the nomogram model was developed based on the regression equation (Figure 4). With the exception of age, which was a continuous variable, other indices, including the SII, serum potassium, responsible artery and PT were binary variables. For SII, 0 represents low SII, and 1 represents high SII. For sex, 0 represents male and 1 represents female. For serum potassium, 0 represents normal and 1 represents hypokalemia. As for the responsible artery, 0 represents the internal carotid and vertebrobasilar artery, and 1 represents the middle cerebral artery. For PT, 0 represents normal PT and 1 represents prolonged PT. The nomogram, calibration curve, and ROC curve were plotted, and the C-index was calculated. Each item in the nomogram had a score and the sum of the scores indicated the risk of DC.

**Figure 5:**
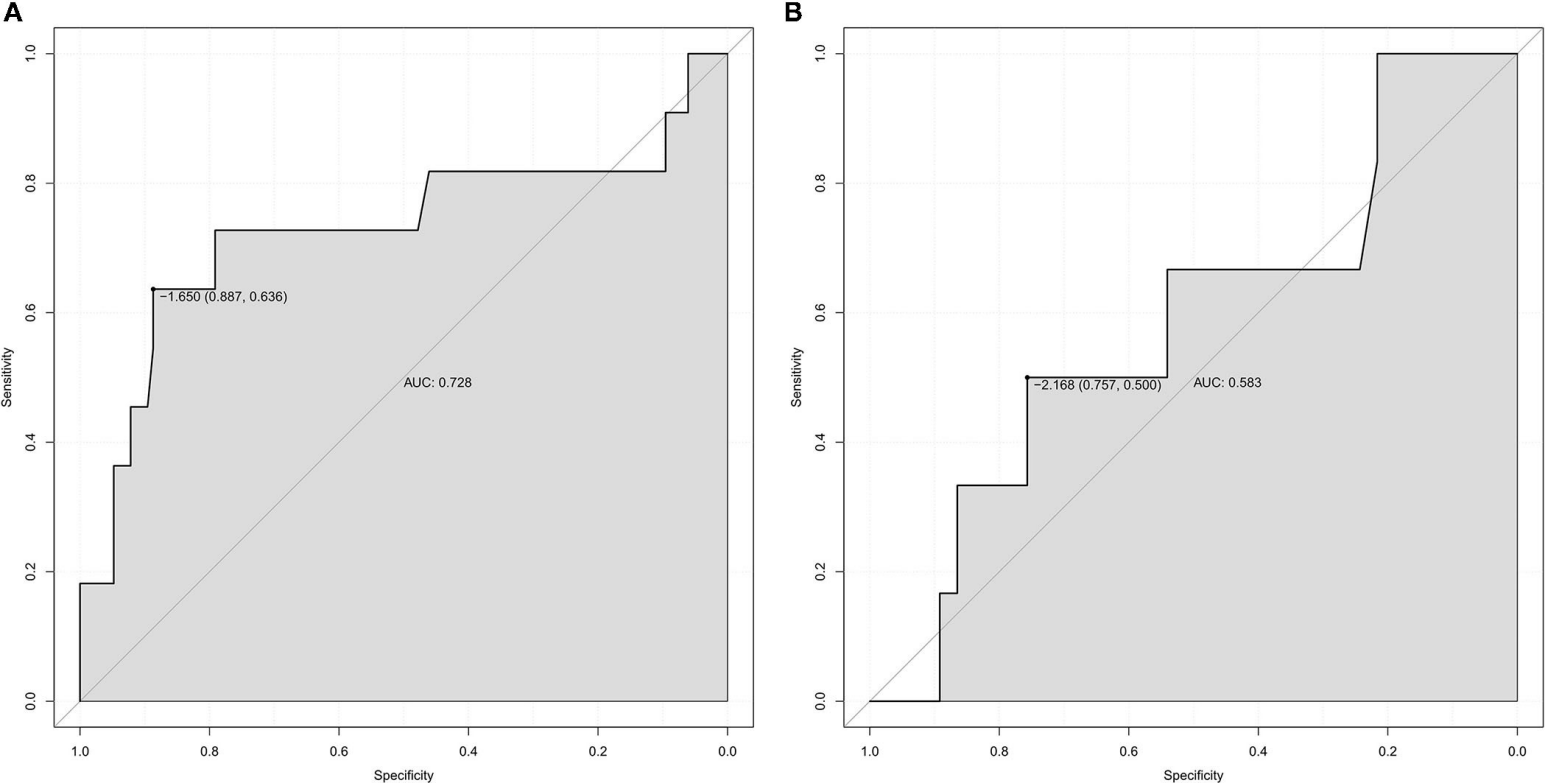
**(A)** ROC curve of training set data. **(B)** ROC curve of testing set data.

**Figure 6:**
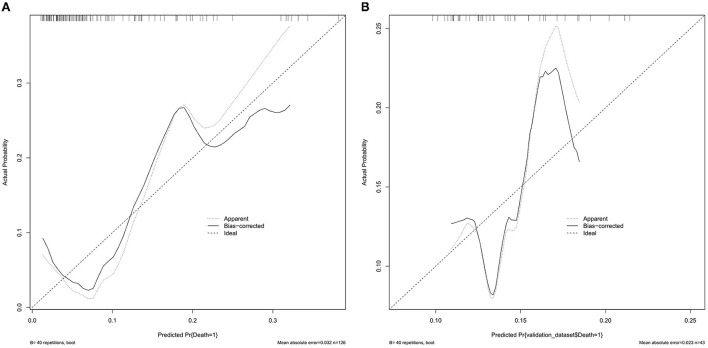
**(A)** Calibration curve of training set data. **(B)** Calibration curve of testing set data.

The AUC of the training set was 0.728 (Figure 5A), and the AUC of the validation set was 0.583 (Figure 5B). The model showed good discrimination and predictive ability. The nomogram was calibrated using a calibration curve, and the training set calibration curve (Figure 6A) suggested that the model had an average error of 0.032 in predicted vs. the actual risk of developing the adverse outcome. Similarly, the validation set calibration curve (Figure 6B) showed that the average error was 0.023, proving that the predictions were in good agreement with the observations. The C-index was 0.804, confirming the accuracy of the nomogram.

## Discussion

In China, the weighted prevalence of stroke is increasing year by year, from 2.28% in 2013 to 2.58% in 2019. People's understanding of stroke is also gradually changing, and more and more studies pay attention to the predictors of stroke (21). The role of inflammation in patients with LAO-AIS after MT remains unclear. Related inflammatory factors such as chemokines can be released in certain brain tissues in patients with AIS, which can aggravate neurological dysfunction (22). Infiltration of leukocytes and various inflammatory mediators can accelerate neuronal apoptosis, thereby aggravating brain injury (23). Neutrophils rapidly migrate to ischemic brain tissues and exacerbate stroke injury by releasing reactive oxygen species, proteases, and pro-inflammatory cytokines (24). Patients with AIS undergo a complex neurohormonal response that induces lymphopenia, leading to disruption of the blood-brain barrier (25). Activated platelets in patients with AIS can interact with T lymphocytes and produce inflammatory factors to slow down the recovery of cranial nerve function (26).

As a new inflammatory marker, the SII has attracted considerable attention in recent years. Previous studies reported that the SII had a good predictive value for the prognosis of myocardial infarction, breast cancer, and small cell lung cancer (27–29). Luo et al. (30) included 76 patients with aneurysmal subarachnoid hemorrhage in their prognostic analysis, and the results showed that the SII at admission was closely related to the 6-month clinical outcome of patients. The study by Chen et al. (31) also showed that the SII was an independent predictor for delayed cerebral ischemia in patients with aneurysmal subarachnoid hemorrhage. Therefore, the purpose of our study was to use the routine blood test results at admission to calculate the SII and other inflammatory indicators, to evaluate the predictive value of the SII for DC in patients with LAO-AIS, and to construct a nomogram to allow doctors to make decisions timely, predictably and rationally.

It is well-known that patients with LAO-AIS can develop cerebral herniation after MT due to increased cerebral edema, cerebral reperfusion injury, or other complications. DC can save these patients' lives; however, the decision to perform DC should be quick and reasonable (32). Our study suggests that the SII is an independent predictor for the requirement of DC in patients with LAO-AIS after MT. However, the nomogram we developed based on patients' clinical data showed good predictive power. The results of routine blood and blood biochemistry tests can be obtained within a few hours after admission and the SII can be calculated immediately. Doctors are able to assess the necessity of DC according to the score of the nomogram and select the best treatment plan.

Our nomogram model showed that older age and lower nomogram scores resulted in a lower probability of undergoing DC. Previous studies have reported that patients with cerebral infarction younger than 60 years old achieved a better outcome after DC (33). We suppose that age is negatively correlated with total brain volume, and elderly people usually have varying degrees of brain atrophy (34); therefore, brain edema after stroke is less likely to cause brain herniation in elderly patients, thereby reducing the probability of DC. Hypokalemia also contributed a certain weight to the nomogram. The study by Wang et al. (35) showed that serum potassium level at admission was associated with stroke recurrence in AIS patients, and some scholars have proposed that emergency stroke patients should be screened immediately for electrolyte disturbances. Early detection and correction of the imbalance of serum potassium improve the prognosis of stroke patients (36). The nomogram we developed showed the predictive power of common clinical factors for DC and can help doctors' decision-making.

There are no relevant studies reporting predictors for DC in patients with LAO-AIS after MT, as well as the construction of a similar predictive model. The SII may be a potential predictor although this needs to be validated by more studies.

The limitations of this study include: (1) The study was a single-center study with a relatively small number of cases, and selection bias may have existed. (2) The study was retrospective, and some confounding factors may be inevitable. (3) There may be differences in the indications of DC among various medical centers, which may affect the statistical analysis, and further multi-center and prospective studies are needed to confirm our results.

## Conclusions

The SII at admission is an independent predictor of the requirement for DC in patients with LAO-AIS after MT. This index can be obtained conveniently and quickly. The SII-based nomogram assists clinicians in assessing the necessity of DC and to make decisions faster and better, so as to improve patients' prognoses.

## Data availability statement

The raw data supporting the conclusions of this article will be made available by the authors, without undue reservation.

## Ethics statement

The studies involving human participants were reviewed and approved by the Ethics Committee of Huizhou Central People's Hospital (kyll2022029). Written informed consent for participation was not required for this study in accordance with the national legislation and the institutional requirements.

## Author contributions

W-CL, Y-XZ, and Z-ZQ conceived the review. GZ, K-LZ, H-YZ, and J-SC conducted data collection. Y-XZ, Y-FD, and Z-ZQ analyzed and interpreted the study data. All authors read and approved the final manuscript.

## Conflict of interest

The authors declare that the research was conducted in the absence of any commercial or financial relationships that could be construed as a potential conflict of interest.

## Publisher's note

All claims expressed in this article are solely those of the authors and do not necessarily represent those of their affiliated organizations, or those of the publisher, the editors and the reviewers. Any product that may be evaluated in this article, or claim that may be made by its manufacturer, is not guaranteed or endorsed by the publisher.
